# High-Definition Transcranial Direct Current Stimulation Over the Right Lateral Prefrontal Cortex Increases Maximization Tendencies

**DOI:** 10.3389/fnbeh.2021.653987

**Published:** 2021-07-19

**Authors:** Haixia Wang, Hanqi Zhang

**Affiliations:** ^1^School of Journalism and Communication and National Media Experimental Teaching Demonstration Center, Jinan University, Guangzhou, China; ^2^School of Economics and Management, South China Normal University, Guangzhou, China; ^3^Key Lab for Behavioral Economic Science & Technology, South China Normal University, Guangzhou, China

**Keywords:** transcranial direct-current stimulation, right dorsolateral prefrontal cortex, maximization tendency, working memory, causal evidence

## Abstract

People seek the best in every aspect of life. However, little is known about the neurobiological mechanisms supporting this process of maximization. In this study, maximization tendencies were increased by using high-definition transcranial direct current stimulation (HD-tDCS) over the right dorsolateral prefrontal cortex (DLPFC). Participants (*n* = 64) received 2 mA anodal 4 × 1 HD-tDCS or sham stimulation over the right DLPFC in two sessions and subsequently completed an N-back working memory task and a maximization scale (MS). We observed that maximization tendency scores increased during anodal stimulation. Furthermore, the results indicate that this increase in maximization tendency was driven by motivational changes. On the MS, alternative search subscale scores were significantly increased, demonstrating an increase in motivation to evaluate more alternatives; however, the results did not indicate that the increase in maximization tendencies was due to working memory improvement. These results demonstrated that maximization tendencies can be strengthened through noninvasive interventions and that the right DLPFC has a causal relationship with maximization tendencies.

## Introduction

People seek the best in every aspect of life. People search for the most suitable schools, jobs, spouses, and more. Striving for the best is synonymous with the pursuit of excellence advocated by the internationally bestselling book *In Search of Excellence* (Peters and Waterman, 1982). The rational choice theory has been applied to explain preference and choice by assuming that people are rational choosers (von Neumann and Morgenstern, 1944). According to the rational choice framework, human beings compare options on a single scale of preference, value, or utility to maximize these qualities (Schwartz et al., 2002).

However, according to Simon (1955, 1956), maximization is often not realizable because of the conflict between the limited cognitive resources of the human brain, such as limited working memory (Baddeley, 1992, 2000) and the unlimited information in the environment. Hence, people often choose an option that is “good enough,” foregoing efforts to maximize choices. Schwartz et al. (2002) expanded on the ideas of Simon, developing the maximization scale (MS) to measure maximization tendencies. The maximization tendency denotes the predisposition of individuals to search for the best option rather than settling for an option that exceeds an internal threshold of acceptability (Simon, 1955, 1956; Schwartz et al., 2002). Schwartz et al. (2002) categorized people as either maximizers who generally search for the best outcome or as satisfiers who settle for a good enough option (Luan and Li, 2017). Since the influential research of Schwartz, studies have investigated maximization tendencies (Iyengar et al., 2006; Chowdhury et al., 2009; Dar-nimrod et al., 2009; Polman, 2010; Cheek and Schwartz, 2016) and have revealed the psychological characteristics of maximizers. Although describing the characteristics of maximization is useful, how to promote maximization tendencies is also a major topic in need of clarification. Yet, few studies have addressed this topic. To the best of our knowledge, no study has produced causal evidence of the neurological processes that encourage humans to strive for the best.

In this study, we explored a method of altering the maximization tendency by applying high-definition transcranial direct current stimulation (HD-tDCS) to investigate the causal mechanisms of maximization. Theoretically, two mechanisms could cause maximization. One mechanism is the limited cognitive resources of the human brain. The cognitive limitation theory (Simon, 1955, 1956) suggests that the most important factor limiting maximization is that humans do not have sufficient cognitive resources to pursue excellence. By contrast, the other possible mechanism is human motivation. People differ in their motivation to invest resources and time in the decision-making process; maximizers are those who are motivated to find the best possible option by seeking information about as many alternatives as possible before making a choice (Lai, 2011). Studies have demonstrated that maximizers expend substantial effort to maximize utility, whereas satisfiers expend less effort to obtain a good enough option (Luan and Li, 2017). These findings suggest that maximizers have greater motivation to pursue the best outcome than satisfiers do. Consequently, there are two approaches to increasing maximization tendencies, by increasing working memory resources or by increasing maximization motivation.

These two possible mechanisms of maximization tendency promotion are both associated with the right dorsolateral prefrontal cortex (DLPFC), and thus, the DLPFC is a primary target for brain stimulation studies aiming to modulate the maximization tendency. First, DLPFC is associated with working memory resources. A study found that modulating the cortical activation of the right DLPFC improves working memory (Wang et al., 2018). A study of lesions also found that the right DLPFC is critical for manipulating information in a broad range of reasoning contexts (Barbey et al., 2013). Second, DLPFC is likely to participate in a network of brain regions involved in motivation processing (Ballard et al., 2011). Empirical evidence has demonstrated that disruptions of the lateral prefrontal cortex by using low-frequency repetitive transcranial magnetic stimulation are correlated with intertemporal self-control (Figner et al., 2010), which suggests that the lateral prefrontal cortex is a crucial neural component of the motivation of self-control. A functional magnetic resonance imaging study demonstrated that activation of the right DLPFC is associated with motivation (Spielberg et al., 2012), and a tDCS study also demonstrated that the DLPFC has a causal relationship with motivation (Kelley et al., 2017; Ohmann et al., 2018).

We present causal evidence for a neural mechanism that regulates maximization. The evidence was collected by applying HD-tDCS to 64 subjects. HD-tDCS is a noninvasive method to modulate neural excitability in healthy humans by applying weak electric currents to the scalp (Nitsche and Paulus, 2011; Woods et al., 2016). Studies have demonstrated that motivation is modulated by applying HD-tDCS to the DLPFC (Kelley et al., 2017; Ohmann et al., 2018) and have demonstrated that working memory increases when the excitability of the right DLPFC is enhanced (Wu et al., 2014; Wang et al., 2018). In accordance with the working memory literature, we predicted that an increase in right DLPFC activity would result in better working memory performance compared with sham stimulation (Wu et al., 2014; Wang et al., 2018), and we also predicted that an increase in right DLPFC activity would result in greater motivation compared with sham stimulation (Kelley et al., 2017; Ohmann et al., 2018), meaning that participants would be willing to allocate more effort to get the best outcome. We thus hypothesized that increasing right DLPFC activity would enhance the maximization tendency through these two possible mechanisms.

## Materials and Methods

### Participants

We used the software G^∗^power to determine sample sizes. The *F*-test was used for data analysis (repeated measures, within factors). For an effect size of *F* = 0.3145 (Partial eta square = 0.09) and power = 0.8, the sample size must be >22. This study had 64 participants, and thus, this study had the necessary statistical power. About 64 healthy students were recruited from Peking University (35 women, 29 men; mean age = 20.36 ± 0.25 years). None of the participants had a history of neurological or psychiatric conditions. All participants provided written informed consent prior to the start of the experiment, which was approved by the Ethics Committee of the School of Psychological and Cognitive Sciences, Peking University. No participants were excluded throughout the experiment.

### High-Definition Transcranial Direct Current Stimulation

**FIGURE 1:**
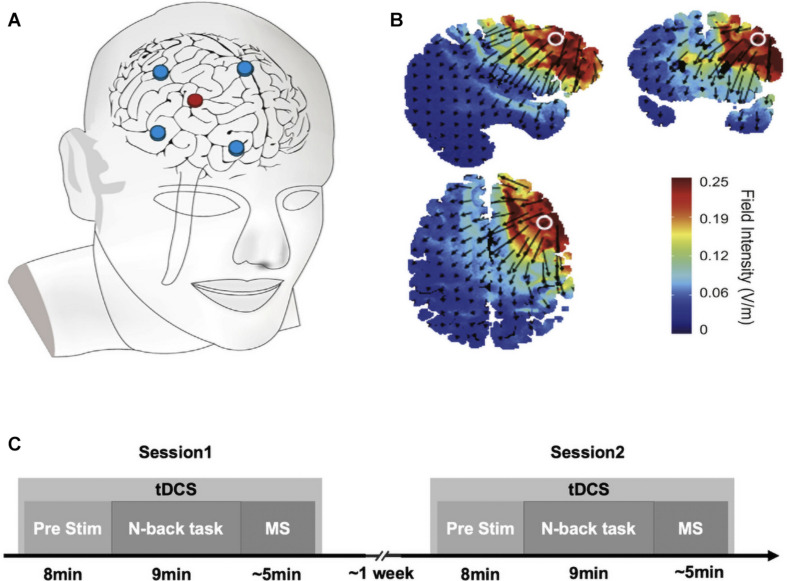
Transcranial direct current stimulation (tDCS) protocols and electric field simulation. **(A)** Electrode placements. **(B)** Electric field simulations. Arrows point in the direction of current flow, and arrow length indicates current flow intensity. **(C)** Experimental procedures.

High-definition transcranial direct current stimulation was delivered using a 4 × 1 adapter (Soterix Medical, 4 × 1-C3, New York) that converted a 2-channel Soterix Medical tDCS stimulator into an HD-tDCS device. Five Ag–AgCl sintered ring electrodes embedded in an EEG cap were held in plastic casings filled with conductive gel and attached to the adaptor device. Each electrode had a contact area of approximately 4 cm^2^ with the skull. The electrodes were arranged on the skull in a 4 × 1 ring configuration (Shen et al., 2016; Figure 1A). For right DLPFC stimulation, the locations corresponded roughly to C4, Fz, Fp2, and FT8, with the central electrode at F4 (Figures 1A,B). The polarity of the current on the target brain area depended on the central electrode. Central anodal stimulation was used for excitatory modulation, and central sham stimulation was used for the control group. For the anodal stimulation, an approximately 0.5 mA/cm^2^ peak current density at the central electrode (F4) was created with a 2.0 mA current, and the peak current density at the return electrodes was roughly 0.125 mA/cm^2^ (C4, Fz, Fp2, and FT8). For anodal stimulation, subjects received a constant current of 2.0 mA at the central electrode with a 30 s ramp-up at the beginning of stimulation and a 30 s ramp-down at the end of stimulation. For sham stimulation, participants only received a 30 s ramp-up at the beginning of stimulation and then a 30 s ramp-down.

### Working Memory

Working memory was measured by using the number N-back task (*N* = 0, 1, 2, 3; 12 trials for each block and four blocks for each N). The number N-back task is a continuous recognition measure that uses long sequences of numbers. For each item in the sequence, the participant must judge whether it matches the number presented N items ago. N is changed between trial blocks (e.g., in the sequence 2-1-2-7-5-3-7, the second “2” would be a 2-back match, and the second “7” would be a 3-back match). The number of correct trials was used as the working memory score.

### Maximization Tendencies

We used the 13-item MS to measure maximization tendencies. Schwartz et al. (2002) validated the MS across several survey and experimental studies. The MS consists of three facet scales. The alternative search facet scale measures the degree to which an individual keeps searching for better alternatives. The decision difficulty facet scale represents the difficulty experienced by the participant when making decisions. The high standards facet scale represents the tendency of decision makers to hold high standards for themselves and in general. MS is a self-reported measurement. Self-reported questionnaire measurements are often used in tDCS experiments (Kelley et al., 2013; Maréchal et al., 2017).

### Experimental Procedure

Participants attended two HD-tDCS sessions between 5 and 8 days apart. The procedure was the same for each session. The stimulation order (anodal tDCS vs. sham tDCS) was counterbalanced across participants to avoid tDCS carry-over effects. After obtaining informed consent, the electrodes were placed on the scalp, the task was explained, participants were instructed to complete a short practice, and then stimulation was applied. Two types of stimulation were performed: anodal (F4+) and sham stimulation. The right DLPFC was localized by using a 10/20 EEG system at F4. Before beginning the task, 8 min of HD-tDCS was applied. We used an online stimulation paradigm, meaning that HD-tDCS was used while participants completed the N-back working memory task and the MS (Figure 1C).

### Statistical Analysis

To test the hypothesis, a one-way ANOVA with repeated measures was used to assess the effects of tDCS (SPSS 20, IBM, Armonk, NY, United States). Order was considered a covariate. Effect sizes were reported as partial eta square ($\eta_{\text{p}}^{2}$). A *p*-value < 0.05 was considered significant. Data are given as mean ± SEM.

## Results

**FIGURE 2:**
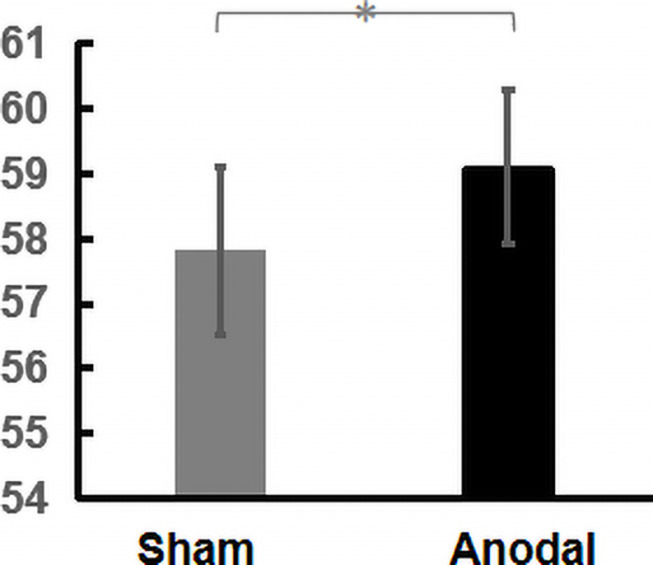
Effect of stimulation on maximization tendency (^∗^*p* < 0.05, error bars represent SEM).

All participants received anodal stimulation or sham stimulation to the right DLPFC across two sessions *via* HD-tDCS. A one-way repeated-measures ANOVA with order as a covariate revealed a significant main effect of HD-tDCS on the maximization scores (MS_*Anodal*_ = 59.08, MS_*Sham*_ = 57.79, *F*_(1,62)_ = 6.354, *p* = 0.014, $\eta_{\text{p}}^{2}$ = 0.093, Figure 2). This result demonstrated that the maximization tendency can be increased through noninvasive interventions and that the right DLPFC has a causal relationship with this tendency.

To test which facet scales of the MS were significantly affected by HD-tDCS, we conducted one-way ANOVA with order as a covariate; this analysis revealed a significant main effect of stimulation on alternative search [*F*_(1,62)_ = 4.127, *p* = 0.046, $\eta_{\text{p}}^{2}$ = 0.062] but no main effect of stimulation on decision difficulty [*F*_(1,62)_ = 2.442, *p* = 0.123] or high standards [*F*_(1,62)_ = 0.176, *p* = 0.676]. The alternative search included items broadly related to the tendency to search for alternatives and to compare among alternatives to identify the best choice (Cheek and Schwartz, 2016). Therefore, anodal HD-tDCS over the right DLPFC increased participant tendencies to search for more alternatives and to compare more alternatives, indicating greater motivation to maximize and supporting the motivation hypothesis.

To examine whether anodal tDCS increases the maximization tendency through the enhancement of working memory, we analyzed the working memory of participants under the influence of tDCS. A one-way ANOVA with order as a covariate revealed a main effect of stimulation on working memory [*F*_(1,62)_ = 38.916, *p* < 0.001, $\eta_{\text{p}}^{2}$ = 0.389]. However, Pearson correlation analyses revealed that working memory was not significantly correlated with the maximization tendency (*r* = −0.134, *p* = 0.295) and working memory was also not significantly correlated with alternative search (*r* = −0.021, *p* = 0.869). Furthermore, changes in working memory were not associated with an increase in the maximization tendency (*r* = −0.042, *p* = 0.746) or alternative search (*r* = −0.083, *p* = 0.516). These results demonstrated that the increase of the maximization tendency after HD-tDCS over the right DLPFC was not caused by an enhancement of executive function. Therefore, these results did not support the cognitive resources hypothesis.

## Discussion

Striving for the best affects every aspect of life. However, remarkably little is known about the neurobiological mechanisms that influence maximization. To answer this question, we investigated whether the maximization tendency can be increased by applying HD-tDCS over the right DLPFC. Participant maximization tendency scores were significantly increased by applying anodal HD-tDCS over the right DLPFC. The right DLPFC has a causal role in modulating the maximization tendency. The performance of working memory and alternative research MS scores were also significantly improved by applying anodal HD-tDCS over the right DLPFC. However, working memory was not associated with the maximization tendency, and improvements in working memory did not explain the increases in the maximization tendency or alternative research MS scores. Therefore, these results did not support the limited cognitive resources hypothesis.

However, the results did support the motivation hypothesis. Specifically, of the three facets of the MS scale, alternative search, decision difficulty, and high standards, motivation to identify the best option was most strongly affected by alternative search tendencies (Schwartz et al., 2002). As predicted, alternative search scores were significantly increased during stimulation, although the same effect was not observed for decision difficulty or high standard scores. Alternative search includes items broadly related to the tendency to search for and compare among alternatives to identify an optimal choice (Cheek and Schwartz, 2016). Thus, increased alternative search scores during anodal HD-tDCS over the right DLPFC suggests participants were more motivated to make optimal choices. Maximizers are more likely to engage in both upward and downward social comparison (Schwartz et al., 2002). Previous studies have demonstrated that activity in the right DLPFC reflects upward social comparison (Lindner et al., 2015). Enhancement of right DLPFC activity might improve satisfier sensitivity to social comparison information, which motivates people to maximize to avoid being viewed as inferior to others. A study demonstrated that greater relative right DLPFC activity is associated with avoidance motivation (Kelley et al., 2017), and therefore, stimulation over the right DLPFC might motivate people to avoid inferior social positions. Studies have also demonstrated that alternative search is correlated with motivation (Kelley et al., 2017; Ohmann et al., 2018). Therefore, applying anodal HD-tDCS over the right DPLFC might increase participant motivation and thereby increase their maximization tendency.

One core assumption of rational economics is that human cognitive resources, including working memory, are infinite. Thus, on this view, cognitive resources are always sufficient to make rational choices; that is, to make the best choice (von Neumann and Morgenstern, 1944). A competing premise is that cognitive resources such as working memory are limited (Simon, 1955, 1956). Thus, people will not always maximize. Psychological and brain imaging studies support this premise (for reviews, as shown in Kahneman, 2011). Based on this evidence, we predicted that improving working memory is an effective approach to increase the maximization tendency by reducing decision-making difficulties. However, in the control condition of this study, working memory was demonstrated to be negatively correlated with decision difficulty (*r* = −0.301, *p* < 0.05) in accordance with previous research (Baddeley, 1992). However, in the experimental condition, working memory had no relationship with maximization, suggesting that working memory may not be an effective approach to improve maximization.

Overall, these results indicate that HD-tDCS over the right DLPFC increases the maximization tendency. The results are consistent with those of studies showing several possible neural mechanisms for how DLPFC influences maximization motivation. More research is necessary to understand which of these mechanisms have effects. Possible mechanisms might be the modulation of activity in motivation regions, input into motivation areas, the differential influence of attention given to alternative options, or influencing maximization choice (such as the effort to search for alternatives; Schwartz et al., 2002). Regardless of the neural mechanism, to the best of our knowledge, the results are the first evidence that DLPFC-related neural processes should be incorporated into existing neural models of choice maximization.

This demonstration of a neurobiological basis for maximization tendencies has crucial implications for therapeutic applications. Moreover, the findings that neural processes can be modulated by using tDCS to influence maximization tendencies may be valuable for increasing the performance and efficiency of individuals (Reardon, 2016).

This study has limitations. First, only the causal role of the right DLPFC in modulating maximization and only two possible mechanisms were investigated in this experiment. Future research could investigate whether modulation of right DLPFC activity increases the maximization tendency through affective processes such as regret and satisfaction. Second, in accordance with previous studies (Kelley et al., 2013; Maréchal et al., 2017), a self-reported questionnaire (MS) was used as a proxy measure of maximization tendencies; however, future research should use a decision-making task to assess whether participants maximize more often after anodal tDCS stimulation over the right DLPFC.

## Data Availability Statement

The raw data supporting the conclusions of this article will be made available by the authors, without undue reservation.

## Ethics Statement

The studies involving human participants were reviewed and approved by the Ethics Committee of the School of Psychological and Cognitive Sciences, Peking University. The patients/participants provided their written informed consent to participate in this study.

## Author Contributions

HZ designed and performed the study and analyzed the data. HW drafted the manuscript. Both authors contributed to the article and approved the submitted version.

## Conflict of Interest

The authors declare that the research was conducted in the absence of any commercial or financial relationships that could be construed as a potential conflict of interest.
